# Growth Hormone Secretion Patterns in German Landrace (DL) Fetuses and Piglets Compared to DL Piglets with Inherited 1,25-Dihydroxyvitamin D3 Deficiency

**DOI:** 10.3390/nu10050617

**Published:** 2018-05-15

**Authors:** Manfred Mielenz, Michael W. Pfaffl, Christina Schlumbohm, Johein Harmeyer, Nahid Parvizi

**Affiliations:** 1 Department of Functional Genomics and Bioregulation, Institute of Farm Animal Genetics, Friedrich-Loeffler-Institut (FLI), Mariensee, 31535 Neustadt a. Rbge., Germany; nahid.parvizi@fli.de; 2 Institute of Nutritional Physiology “Oskar Kellner”, Leibniz Institute for Farm Animal Biology (FBN), Wilhelm-Stahl-Allee 2, 18196 Dummerstorf, Germany; 3 Department of Animal Physiology and Immunology, Technical University Munich, Weihenstephaner Berg 3, 85354 Freising, Germany; michael.pfaffl@wzw.tum.de; 4 Department of Physiology, University of Veterinary Medicine Hannover, Foundation, Bischofsholer Damm 15, 30173 Hannover, Germany; cschlumb@gmx.de (C.S.); Johein.Harmeyer@tiho-hannover.de (J.H.); 5 Neu Encepharm GmbH, Hans-Adolf-Krebs Weg 9, 37077 Göttingen, Germany

**Keywords:** growth hormone, growth hormone receptor, pig, fetus, piglet, vitamin D, vitamin D deficiency

## Abstract

The regulation of growth hormone (GH) release during prenatal development and during early postnatal life is not entirely clarified. In this study plasma GH concentrations in pigs with inherited pseudo vitamin D deficiency type I (PDDR-I), which regularly show growth retardation, were compared during ontogeny with unaffected pigs of the same breed (German Landrace, DL) as control. Plasma GH concentrations were measured in plasma of chronically catheterized fetuses (beginning on day 101 after mating or after artificial insemination) and in piglets (day 37 postpartum (p.p.)—day 42 p.p.) of both lines. A growth curve beginning at day 7 p.p. was recorded for both lines. The relative amount of GH receptor (GHR) mRNA in liver was quantified by competitive reverse transcription polymerase chain reaction in piglets at day 42 p.p. A trend for higher GH concentrations was observed in PDDR-I fetuses (*p* < 0.1). In PDDR-I piglets compared to DL piglets higher plasma GH values (*p* < 0.01), were observed despite lower body weight. The relative quantity of GHR mRNA in liver was not significantly different between the two lines. Piglets with an inherited defect of vitamin D synthesis showed higher GH concentrations. A hormonal imprinting by low 1,25(OH)_2_D_3_ could be one reason for our observations and should be analysed in detail in future.

## 1. Introduction

Inadequate vitamin D sustenance is currently a global problem in humans [1]. Deficits depend for example on the exposition to sunlight, affecting also diseases beyond calcium metabolism, as discussed by Sowah et al. [2]. Lower birth weight and disturbed neonatal growth in humans are associated with maternal vitamin D deficiency [3,4,5]. Recently, it was suggested that vitamin D treatment may improve the management of GH deficiency in humans because it increases the secretion of circulating insulin-like growth factor-I (IGF-I) [6]. It is generally accepted that growth hormone (GH) is active at all stages of development and that GH-deficient newborns have abnormal growth pattern [7]. However, in late gestation growth is mostly related to hormones influencing maternal nutrient partition via placental blood flow to the fetus [8]. In newborn piglets it was shown that the functional somatotropic axis is stimulated rather by nutritional stimuli than by GH [9]. The regulation of body weight in piglets is not directly linked with GH secretion up to 40 days p.p. because the concentrations of GH were not different between heavy an light male piglets of a litter [10]. However, the GH receptor (GHR) mRNA abundance is linked with the nutritional status of the piglet. Fasting for 20–24 h reduces the amount of GHR mRNA in the liver of 7 weeks old piglets [11]. The IGF-I response to recombinant porcine GH challenge is only moderate up to day 37 postpartum (p.p.) but increases progressively afterwards [12].

Also, piglets with inheritable pseudo vitamin D deficiency type I (PDDR-I) show reduced body weight [13]. PDDR-I pigs have a defect of renal 25-hydroxyvitamin D_3_ 1α hydroxylation due to dysfunctional 1α-hydroxylase (CYP27B1) [14,15]. Deficiency in 1,25(OH)_2_D_3_, the most active form of vitamin D, has no impact on the transplacental transfer of calcium and phosphorus homeostasis in pigs [16]. Nevertheless, as reviewed by Eyles et al. [17] vitamin D deficiency influences brain development and function in general. Among others, an impairment of the ontogeny of dopamine neurons was observed [18]. The regulation of different genes like catechol-o-methyltransferase, involved in the differentiation of dopaminergic neurons, is affected [19]. Dopaminergic pathways are involved in GH release [20]. As a result, GH secretion might be influenced by vitamin D deficiency.

Based on the mentioned relationships, we aimed to compare plasma GH concentrations in German Landrace (DL) fetuses and piglets with their PDDR-I counterparts. To exclude potential effects linked with deficits in nutrient intake over time, we compared the GHR mRNA abundance in liver of both lines.

## 2. Materials and Methods

All experimental and surgical procedures were in accordance with procedures approved by the regional Animal Ethics Committee (AZ: 604i-42502-96/863). Experiments were conducted in fetuses and piglets with a defect in 25-hydroxyvitamin D_3_ 1α hydroxylation (PDDR-I) in comparison to unaffected pigs of the same breed (DL). It is known that PDDR-I piglets show a reduction of 60% to 80% in 1,25(OH)_2_D_3_ plasma levels at weaning [13]. Homozygote parents were mated to get homozygote F1 offspring. The age of the fetuses was referred to the day of mating or artificial insemination as day zero post coitum (p.c.). Animals were bred at the institute’s own animal house and were fed with standard diets for sows. The vitamin D content of any commercial pig diet does not cover the requirement of PDDR-I sows or piglets. To avoid clinical symptoms of vitamin D deficiency during early gestation, pregnant sows were kept on a continuous therapy with vitamin D_3_ (D_3_ Vitamin, water soluble, WDT, Garbsen, Germany). High doses of Vitamin D_3_ in aqueous solution were injected every 6 weeks according to a treatment schedule for PDDR-I pigs (Institute for Physiology, University of Veterinary Medicine Hannover, Foundation, Germany) to reach concentrations 20 to 100 times above the physiological requirement of 8 µg to 10 µg/day [21,22]. The vitamin D_3_ supplementation of the PDDR-I sows was ceased 8 weeks prior term. The piglets had free access to water and were fed ad libitum a piglet starter diet. Piglets did not receive any injection with vitamin D_3_ during the study period.

Plasma GH concentrations of three DL fetuses (2 male, 1 female) and four PDDR-I fetuses (3 male, 1 female), one per sow, were compared. The chronic catheterization of the fetal vena jugularis was performed at day 99 p.c., however, in one animal at day 94 p.p. as described previously [23]. In brief, during lateral laparotomy under general anesthesia one uterine horn was exteriorized. After opening of the uterus, a silicone catheter was placed into the fetal jugular vein and fixed. Thereafter, the uterine incision was closed, and the uterus was placed back into the abdominal cavity. The catheter was routed outside to the maternal back by tunneling using a trocar. Blood sampling started at day 101 p.c. and was repeated every two days as long as the catheter was patent. To control the physiological status of the fetus, blood gas parameters (pO_2_, pH) were analysed (Ciba Corning Diagnostics, Fernwald, Germany) at the beginning and at the end of the blood sampling period. During a two-hour period 12 blood samples were drawn in 15 min intervals but with a frequency of 5 min between 30 and 50 min after the onset of the sampling period. Only blood profiles from fetuses with an oxygen partial pressure of equal or more than 18 mm Hg and a pH value between 7.3 and 7.5 were used for further analysis.

German Landrace (*n* = 8; 4 male, 4 female) and PDDR-I piglets (*n* = 7; 5 male, 2 female) from two sows of each line were used to compare GH values. They were weaned at four weeks p.p. and kept in groups of five on straw. The body weight of the piglets was recorded from day 7 p.p. up to day 35 p.p. every week. At day 35 p.p. the piglets were provided with chronic catheters in the vena jugularis externa [24]. Blood sampling started two days later. For this, animals were kept in individual cages. Blood sampling at each time point was performed for 120 min in total with a frequency of 15 min but with a frequency of 5 min between 30 min and 50 min after the start of sampling.

Blood samples from fetuses and from piglets were transferred to heparinized tubes at each indicated time point and stored on ice up to the end of the sampling period. Plasma was stored at −20 °C until analysis. After the sampling period, the piglets were relocated to their groups. The piglets were slaughtered at 42 days p.p. at the institute’s own slaughterhouse and liver samples of four male and four female piglets of each line were collected. The samples were immediately frozen in liquid nitrogen and stored at −80 °C until RNA extraction.

Blood sampling in fetal pigs and piglets started at 9.00 a.m. and tissue sampling in piglets was performed after feeding in the morning (approx. 6.00 a.m.) within the same time period of 2–3 h.

GH measurement was performed with a homologous double antibody radioimmunoassay in accordance to Bauer & Parvizi [23]. Porcine GH (Biogenesis, Poole, UK) was used for labeling and for standards. The intra-assay variance was 9% and the inter-assay variance was 14%. Samples were analyzed in duplicate.

Relative GHR mRNA quantification was performed according to the method we published previously [25]. In brief, 1.8 µg total RNA was reverse transcribed with superscript II RNAse H^−^ (Life Technologies, Gaithersburg, MD, USA) and random hexamer primers (2.5 µM, PE, Applied Biosystems, Foster City, CA, USA) in a volume of 20 µl using water instead of total RNA as negative reverse transcription control (-RT). -RT controls were run in each quantitative polymerase chain reaction assay as negative controls. Relative quantification of porcine GHR mRNA (National Institutes of Health acc. no.: NM214254) was done by competitive PCR (Clontech Laboratories, Palo Alto, CA, USA). Amplification was performed in a total volume of 50 µL with 3 µL cDNA; 0.4 µM of the following intron-exon spanning primers were used (forward: 5′TGAGCCCATTTGCATGTGAAG′3; reverse: 5′TCTGAGCCTTCAGTCTTTTCATC′3). The resulting GHR PCR products (322 base pairs) and the corresponding competitor (431 base pairs) were visualized by ethidium bromide on agarose gels. PCR products were quantified densitometrically (Image Master ID Elite, V3; Pharmacia Biotech, Uppsala, Sweden). The linearity at different cycles was tested [25]. The measured concentrations at different cycle numbers were not different. Finally, the GHR cDNA and its competitor were amplified by PCR with 28 cycles. The log ratio between the GHR PCR product and its competitor was expressed (coefficient of variation: 7.4%). Normalization was done against the stable expressed reference gene 18 S rRNA (18 S rRNA primers: Ambion, Austin, TX, USA), which was amplified for 16 PCR cycles, and quantified accordingly.

The GH plasma concentrations were analyzed by the mixed procedure of SAS (ver. 9.4, SAS Institute Inc., Cary, NC, USA) for repeated measures. Group, time and their interaction were used as fixed effects. For the fetal data the first-order autoregressive covariance structure and for piglets ante-dependence were used as covariance structures. Multiple comparisons were tested by the Tukey-Kramer test. If more than one blood profile of a sampling period of 2 h on different days was taken from one animal, the mean of each time point was used for statistical analysis. Student’s *t*-test was used for analysis of the GHR mRNA data in DL piglets. Due to limited and unbalanced data of both sexes, data were merged. Results were shown as means ± standard error of mean (SEM). Statistical significance was considered for *p* < 0.05 and a trend for *p* < 0.1.

## 3. Results

**Figure 1:**
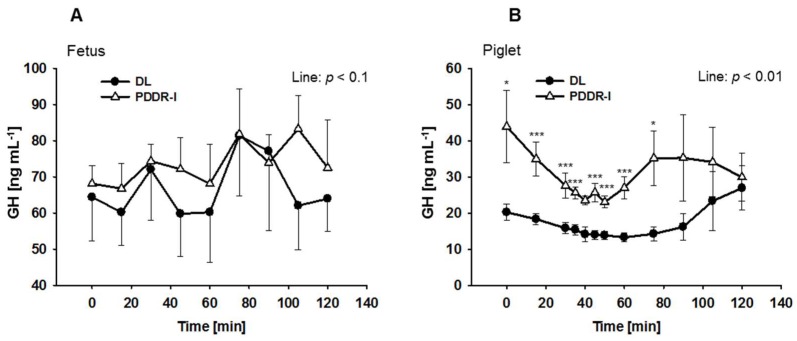
Growth hormone (GH) plasma concentrations in chronically catheterized German Landrace (DL) and pseudo vitamin D deficiency type I (PDDR-I) fetuses and piglets. (**A**) GH plasma concentrations in fetuses. Data represent the means of 8 blood profiles of DL (*n* = 3) and the means of 14 blood profiles of PDDR-I fetuses (*n* = 4). The samples were collected within 1 to 4 sampling days after catheterization. (**B**) GH plasma concentration in piglets. Piglets were catheterized at 35 days postpartum (p.p.) and blood sampling started at 37 p.p. Concentrations were higher in PDDR-I (*n* = 7) vs. DL piglets (*n* = 8). Sampling was performed for 120 min every 15 min in fetuses and piglets, but with a 5 min frequency between 30 min and 50 min after the start of sampling only in piglets. Data are presented as means ± standard error of mean; * *p* < 0.05, *** *p* < 0.001.

Comparing the mean GH plasma values between eight blood profiles achieved from three DL fetuses and 14 blood profiles obtained from four PDDR-I fetuses, no significant difference between the two lines were observed (*p* < 0.1; in mean: 68.71 ± 3.8 ng mL^−1^ in DL vs. 74.20 ± 2.77 ng mL^−1^ in PDDR-I fetuses) (Figure 1A). The blood profiles were obtained between two days and 10 days after the surgery. Blood gas analysis showed O_2_ values of 21.86 mm Hg (±0.77 SEM) at the beginning and 19.44 mm Hg (±0.74 SEM) in mean at the end of the 120 min sampling period. However, GH plasma concentrations in PDDR-I piglets were higher than in DL piglets between day 37 p.p. and day 42 p.p. (*p* < 0.01; in mean: 30.5 ± 1.8 ng mL^−1^ in PDDR-I vs. 17.0 ± 1.0 ng mL^−1^ in DL piglets) (Figure 1B).

**Figure 2:**
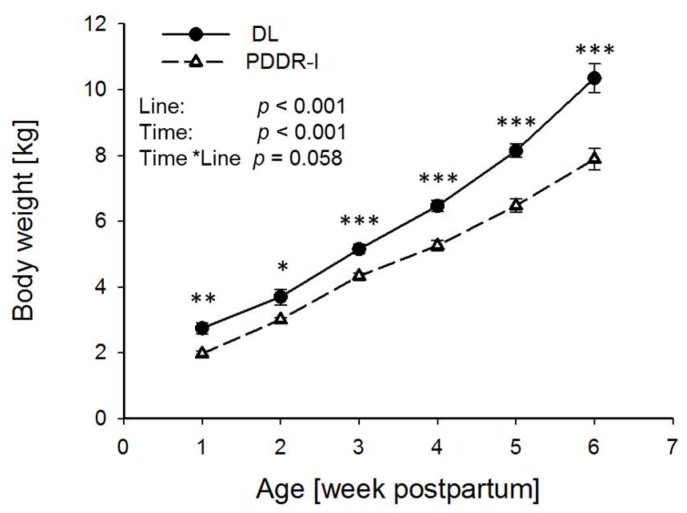
Growth curve of DL (*n* = 8) and PDDR-I piglets (*n* = 7) from week 1 p.p. to week 6 p.p. Data are presented as means ± SEM; * *p* < 0.05, ** *p* < 0.01. *** *p* < 0.001.

Comparing the body weight of both lines between day seven and day 42 p.p. we observed higher body weight in DL piglets (*p* < 0.001) (Figure 2).

The measurement of GHR mRNA abundance in the liver at 42 days p.p. revealed no significant differences between the piglets of both lines (*p* = 0.96).

## 4. Discussion

Deficiency of 1,25(OH)_2_D_3_ in humans is caused by different factors including the place of residence and lifestyle. Vitamin D deficiency affects birth weight negatively and is associated with disease risks in humans [4,26]. The present study aimed to analyze if there is any relationship between low 1,25(OH)_2_D_3_ synthesis and GH concentrations in blood plasma of the porcine fetus during late pregnancy and piglets within the 6th week of life. In piglets we also analyzed GHR mRNA abundance in liver by competitive PCR as a reliable method [27]. Deficiency of 1,25(OH)_2_D_3_ in PDDR-I piglets has been shown in several previous studies [21,22,28] but was not measured in the current trial. Retrospectively, this appears to be a disadvantage of the present study. However, we observed that the body weight of PDDR-I piglets at each time point analysed was lower than that of the DL piglets. Growth retardation in the offspring of vitamin D deficient mothers is a typical observation [5,29]. Also in newborn PDDR-I piglets lower body weight was reported before [13].

We compared plasma GH concentrations in fetuses of both lines and found no significant disparities. We observed high GH concentrations in fetal pigs that were in the range to those reported by Klindt and Stone 1983 [30]. In neonatal pigs GH declines up to two weeks p.p., increases between the 3rd and 5th week sharply and decreases with advancing age [31]. In the line of these findings we observed relatively high GH plasma concentration in piglets of both lines.

Remarkably, the plasma GH concentrations were greater in PDDR-I piglets than in DL piglets but differences in liver GHR mRNA content were not evident. Dauncey et al. [11] observed that in piglets a relatively small reduction in feed intake, which does not affect growth, is able to decrease GHR mRNA in liver. We observed no decrease in GHR mRNA. Consequently, we ruled out that our observations were related to malnutrition in PDDR-I piglets, showing a lower body weight.

This apparent controversy between lower body weight and higher GH concentrations in piglets may reflect a delayed maturation of central nervous regulatory mechanisms, responsible for the regulation of GH secretion in PDDR-I piglets. Recently it was demonstrated that vitamin D deficiency is associated with changes in different transmitter systems of the neonatal rat [32]. Intrauterine growth restriction in pigs is associated with an increase in brain dopaminergic activity [33], which leads in consequence to a reduced somatostatin tonus on growth hormone releasing hormone secretion and therefore to higher GH concentrations at least in men [34]. As discussed by Eyles et al. [35] vitamin D is needed for the development of the central nervous dopamine system and influences dopaminergic activity in the newborn rat as an outcome of hormonal imprinting [36]. 

## 5. Conclusions

Hormonal imprinting by low 1,25(OH)2D3 concentrations could be one reason for significantly higher GH concentrations in PDDR-I piglets and should be analysed in detail in future.
